# Diagnostic Potential of Multidetector Computed Tomography for Characterizing Small Renal Masses

**DOI:** 10.1155/2015/476750

**Published:** 2015-04-09

**Authors:** Maria Elisabetta Mancini, Annamaria Albergo, Marco Moschetta, Mariacristina Angelelli, Arnaldo Scardapane, Giuseppe Angelelli

**Affiliations:** ^1^Interdisciplinary Department of Medicine (DIM), Section of Diagnostic Imaging, Aldo Moro University of Bari Medical School, Piazza Giulio Cesare 11, 70124 Bari, Italy; ^2^SSFO, U.O. Hospital Pharmacy, University Hospital Policlinico of Bari, Piazza Giulio Cesare 11, 70124 Bari, Italy

## Abstract

*Objectives*. To assess the potential of CT for characterizing small renal tumors. *Methods*. 76 patients with <4 cm renal tumors underwent CT examination. The following parameters were assessed: presence of calcifications, densitometry on unenhanced and enhanced scans, washout percentage, urinary tract infiltration, star-shaped scar, and paradoxical effect. *Results*. Calcifications were found in 7/56 (12.5%) carcinomas. Clear cell carcinomas were as follows: mean density 183.5 HU (arterial phase), 136 HU (portal phase), and 94 HU (delayed phase), washout 34.3%; chromophobe carcinomas were as follows: mean density 135 HU (arterial phase), 161 HU (portal phase), and 148 HU (delayed phase), washout 28%; papillary carcinomas were as follows: mean density 50.3 HU (arterial phase), 60 HU (portal phase), and 58.1 HU (delayed phase), washout 2.7%. In 2/56 (3.6%) cases urinary tract infiltration was found. Oncocytomas were as follows: mean density 126.5 HU (arterial phase), 147.5 HU (portal phase), and 115.5 HU (delayed phase), washout 28.6%. On unenhanced scans, angiomyolipomas were as follows: density values <30 HU in 12/12 (100%) of cases and on enhanced scans: mean density 78 HU (arterial phase), 128 HU (portal phase), and 80 HU (delayed phase), washout 50%. *Conclusions*. Intralesional calcifications and urinary tract infiltration are suggestive for malignancy, with the evidence of adipose tissue for angiomyolipomas and a modest increase in density with a reduced washout for papillary carcinomas. The intralesional density on enhanced scans, peak enhancement, and washout do not seem significant for differentiating clear cell, chromophobe carcinomas, angiomyolipomas, and oncocytomas.

## 1. Introduction

In recent years, the wider use of imaging methods such as ultrasound (US), computed tomography (CT), and magnetic resonance imaging (MRI) in the study of abdominal diseases has led to an increase in the incidental finding of small renal expansive processes, with a prevalence of more than 71% [1].

After recognizing a renal expansive process, differential diagnosis of cystic from solid lesions is crucial for the choice of therapy, and in the case of solid lesions, it is essential to differentiate benign from malignant ones [2–4].

Cystic lesions are generally easy to differentiate because no enhancement is detected after the intravenous injection of contrast medium, while it is generally difficult to characterize solid lesions at CT and the results so far reported are widely discordant [5–9].

In particular, the difficulties of differential diagnosis concern the differentiation among carcinomas, oncocytomas, and angiomyolipomas, which represent the most common benign lesions.

The aim of this research is to assess the potential of CT for characterizing small renal tumors.

## 2. Methods

In the period between January 2010 and March 2014, CT images of 76 patients (male/female, 50/26; mean age 53; range 32–78 years) with an incidental finding of small renal mass were retrospectively evaluated.

Patients underwent CT examination in elective or urgent setting for different clinical problems and without a significant urinary indication in all cases.

The list of the enrolled patients was sought in our database, by using keywords such as “expansive renal lesion.” Thus a list of 220 lesions was obtained; simple renal cysts, cyst-like lesions, solid renal lesions with a diameter greater than 4 cm, and all renal lesions observed in patients with a known primary tumor because of suspected secondary localizations were excluded.

The examined series included 56 renal cell carcinomas (44 clear cell carcinomas, 4 chromophobe carcinomas, and 8 papillary carcinomas), 12 angiomyolipomas, and 8 oncocytomas.

The definitive diagnosis was based on histological control after surgery in case of carcinomas and oncocytomas, while in case of angiomyolipomas it was based exclusively on the densitometric morphology of the lesions.

CT examinations were performed by using 16-row (Aquilion 16, Toshiba Medical Systems, Tochigi, Japan) and 320-row (Aquilion One, Toshiba Medical Systems, Ottawa, Japan) devices. The following parameters were used for 16-row CT: thickness 1 mm; increment 0.8 mm; rotation time 1 s; kV/mAs 120/250; matrix 512 × 512, and the following parameters for 320-row CT: thickness 0.5 mm; increment 0.5; rotation time 0.5 s; kV/mAs 120/250; matrix 512 × 512.

CT scans were acquired before and after intravenous injection of contrast medium (Iomeron 400, Bracco, Milan, Italy; Ultravist 370, Bayer, Berlin, Germany) at a flowrate of 3–3.5 mL/s and a dose of 1.5 mL/kg of body weight. A triphasic technique was used in all cases with image acquisition in the arterial (30–35 s mean delay), portal (65–70 s mean delay), and delayed (240 s mean delay) phases.

All MDCT data were transferred to a workstation (HP XW 8600) equipped with dedicated software (Vitrea FX 2.1, Vital Images, Minneapolis, Minnesota, US) for image reconstructions. Multiplanar (MPR) and volume rendering (VR) reconstruction programs were used. The entire image analysis was performed within 15 min per patient. Axial and reconstructed images were examined in consensus by two experienced radiologists in the field of CT examination of the urinary tract.

The following parameters were considered for evaluating CT images obtained before the intravenous injection of contrast medium:

- presence of calcifications,
- evidence of adipose tissue as markedly hypodense intralesional areas with Hounsfield Unit (HU) values of less than –30,
- densitometric values within the detected lesion.

After the intravenous injection of contrast medium, the following parameters were considered:

- lesion morphology, size, and homogeneous or inhomogeneous appearance;
- mean densitometric values in the arterial, portal, and delayed phases, as assessed in the lesion site and in the intact contiguous renal parenchyma;
- evidence of the “paradoxical effect,” in case of a densitometric increment in the delayed phase within intralesional areas which resulted in being hypodense in the arterial phase;
- washout relative percentage value obtained by dividing the maximum density after the intravenous injection of contrast medium with that in the delayed phase;
- presence of an intralesional star-shaped scar;
- morphology of renal calyces and pelvis in relation to a possible compression or infiltration.

The density values were quantified in HU and calculated by using a 3 mm region of interest (ROI) positioned outside of any areas of intralesional colliquation.

## 3. Results

**Figure 1:**
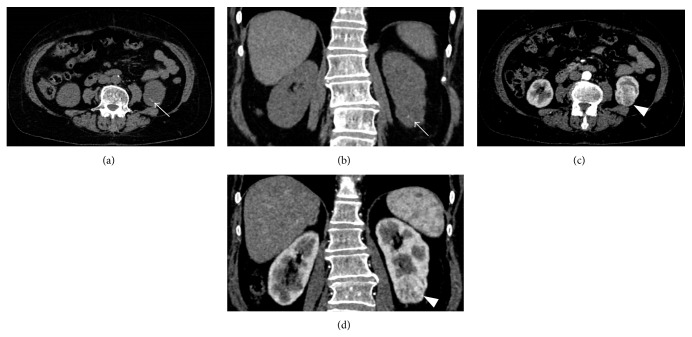
Clear cell carcinoma. ((a)-(b)) Unenhanced CT scans. Small calcifications are recognizable at the lower pole of the left kidney (arrows). ((c)-(d)) Contrast-enhanced CT scans. The lower polar tumor is well evident (arrowheads).

In unenhanced scans calcifications were detected in 7/56 (12.5%) renal cell carcinomas (Figure 1) but never in oncocytomas and angiomyolipomas.

With regard to the intralesional densitometry, HU negative values significant for the presence of intralesional adipose tissue were detected in 12/12 (100%) angiomyolipomas and never in carcinomas and oncocytomas.

In unenhanced scans, 52/56 carcinomas showed higher densitometric values as compared with the contiguous renal parenchyma; in particular, 44/44 clear cell carcinomas had higher values by 4–16 HU (average 10 HU), 4/4 chromophobe cell carcinomas were higher by 3–5 HU (average 4 HU), and 4/8 papillary carcinomas were higher by 4–6 HU (average 5 HU).

On the contrary, in case of oncocytomas, the densitometric unenhanced values resulted in being lower than those of the contiguous renal parenchyma in all cases, with a difference ranging between 3 and 6 HU (average 4.5 HU).

After the intravenous injection of contrast medium, carcinomas had a diameter ranging between 22 and 40 mm, oncocytomas between 20 and 35 mm, and angiomyolipomas between 15 mm and 30 mm.

**Figure 2:**
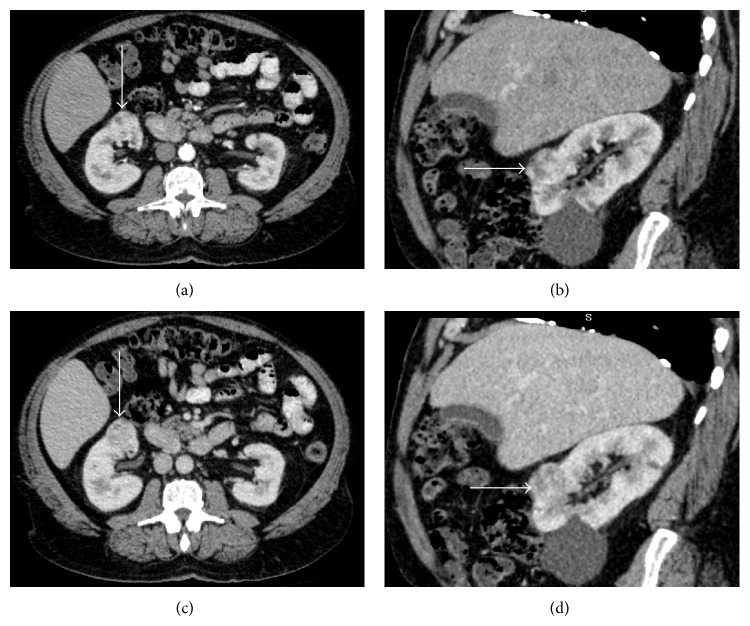
Clear cell carcinoma. ((a)-(b)) Contrast-enhanced CT scans in arterial phase. ((c)-(d)) Portal phase CT scans. The tumor with inhomogeneous aspect is recognizable at the anterior portion of the right kidney (arrows).

**Figure 3:**
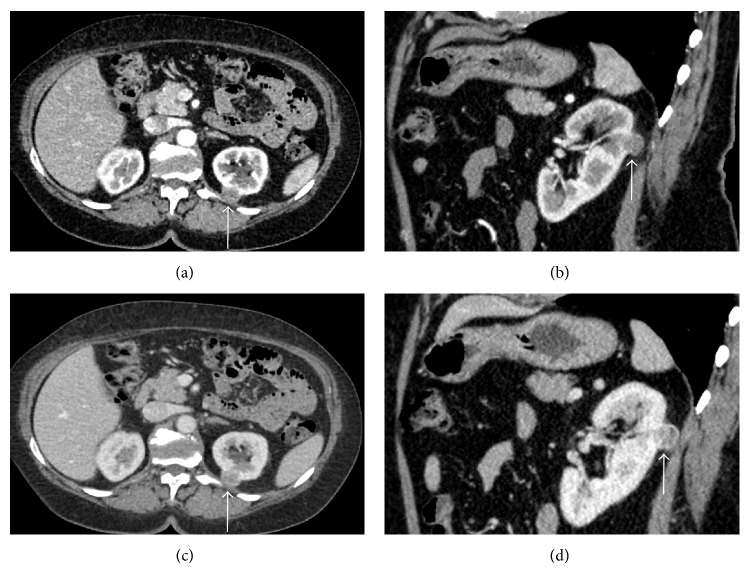
Oncocytoma. ((a)-(b)) Contrast-enhanced CT scans in arterial phase. ((c)-(d)) Portal phase CT scans. A small tumor with inhomogeneous aspect is recognizable at the anterior posterior portion of the left kidney (arrows).

An inhomogeneous intralesional density was recognized in 35/56 carcinomas (62.5%) (Figure 2), in 6/8 oncocytomas (75%) (Figure 3), and in 8/12 (66.6%) angiomyolipomas.

The analysis of the intralesional densitometric changes after the intravenous injection of contrast medium shows that clear cell carcinomas had a peak enhancement in the arterial phase and chromophobe carcinomas, papillary carcinomas, and oncocytomas in the portal phase.

In particular, in case of clear cell carcinomas, density in the arterial phase ranged between 99 and 268 HU (mean value, 183.5 HU), in the portal phase between 93 and 179 HU (mean value, 136 HU), and in the delayed phase between 79 and 109 HU (mean values, 94 HU) (Figure 2).

**Figure 4:**
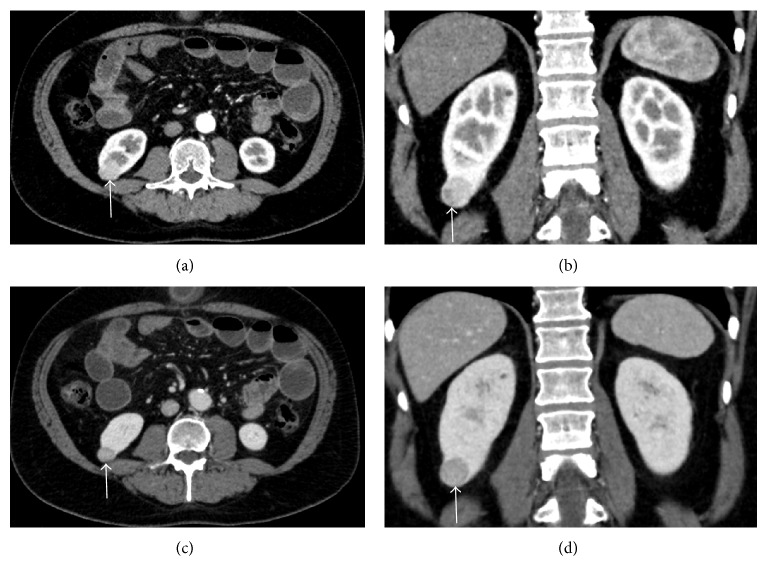
Chromophobe carcinoma. ((a)-(b)) Contrast-enhanced CT scans in arterial phase. ((c)-(d)) Portal phase CT scans. A small tumor with nonspecific morphology is recognizable at the lower pole of the right kidney (arrows).

In case of chromophobe carcinomas, density in the arterial phase ranged between 170 and 190 HU (mean value, 180 HU), in the portal phase between 205 and 215 HU (mean value, 210 HU), and in the delayed phase between 202 and 210 HU (mean value, 206 HU) (Figure 4).

**Figure 5:**
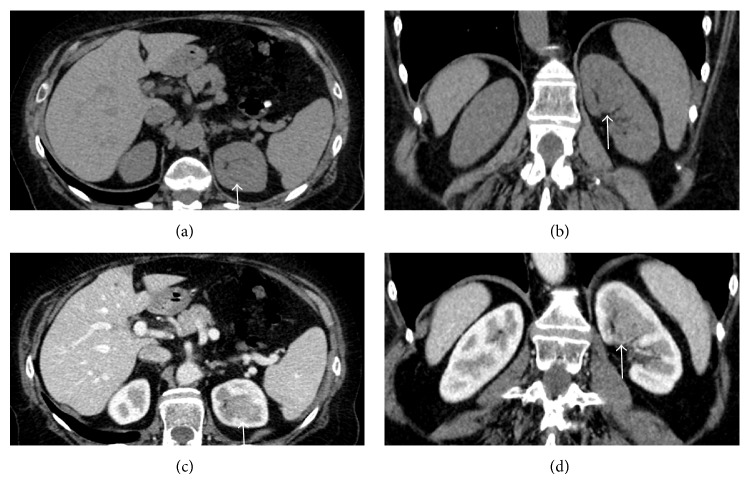
Papillary carcinoma. ((a)-(b)) Unenhanced CT scans. ((c)-(d)) Contrast-enhanced CT scans. A mass with a modest enhancement after contrast medium injection is evident at the upper portion of the left kidney (arrows).

In case of papillary carcinomas, density in the arterial phase ranged between 46 and 54.5 HU (mean value, 50.3 HU), in the portal phase between 52.3 and 67.6 HU (mean value, 60 HU), and in the delayed phase between 51.9 and 64.3 HU (mean value, 58.1 HU) (Figure 5).

In case of oncocytomas, density in the arterial phase ranged between 59 and 170 HU (mean value, 114.5 HU), in the portal phase between 92 and 203 HU (mean value, 147.5 HU), and in the delayed phase between 74 and 157 HU (mean value, 115.5 HU) (Figure 3).

In case of angiomyolipomas, density in the arterial phase ranged between 71 and 86 HU (mean value, 78 HU), in the portal phase between 100 and 156 HU (mean value, 128 HU), and in the delayed phase between 50 and 110 HU (mean value, 80 HU).

Based on the reported data, therefore, the peak of enhancement was detected in the arterial phase in clear cell carcinomas (183.5 versus 136 HU) and in the portal phase in chromophobe cell carcinomas (180 versus 210 HU), papillary carcinomas (50.3 versus 60 HU), oncocytomas (114.5 versus 147 HU), and angiomyolipomas (78 versus 128 HU).

The “paradoxical effect” has been recognized in a single case of oncocytoma and in no case of carcinoma or angiomyolipoma.

The mean washout values in clear cell carcinomas ranged between 20.1% and 48.5% (mean value, 34.3%), in chromophobe carcinomas between 20% and 36% (mean value, 28%), in papillary carcinomas between 0.4% and 5% (mean value, 2.7%), in oncocytomas between 19.5% and 37.7% (mean value, 28.6%), and in angiomyolipomas between 40% and 60% (mean value, 50%).

No case of intralesional star-shaped scar was found in the examined series.

**Figure 6:**
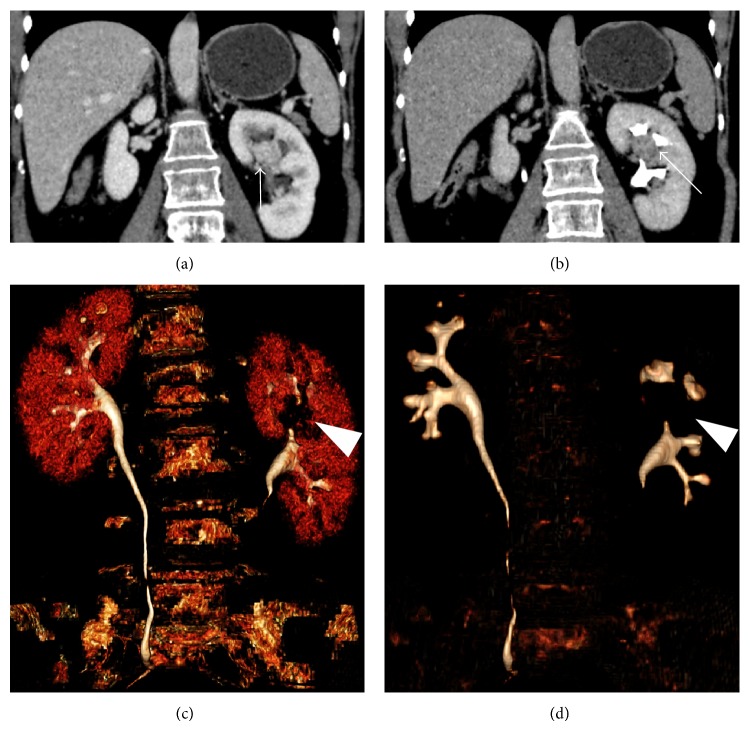
Clear cell carcinoma. (a) Coronal reconstructions in portal phase and (b) in delayed phase. ((c)-(d)) 3D reconstructions. A small tumor (arrows) that infiltrates the urinary tract is recognizable at the upper portion of the left kidney (arrowheads).

Finally, with regard to the infiltration of the urinary tract, it has been recognized only in two cases of clear cell carcinoma (3.6%) (Figure 6).

## 4. Discussion

In recent years, the ever wider use of ultrasound, CT, and MRI has greatly increased the incidental finding of small solid renal lesions.

Different factors are involved in the therapeutic management of such lesions; they are represented above all by their benign or malignant nature and then by size, patient's age, life expectancy, and presence of comorbidities [3, 4].

Among benign lesions, especially angiomyolipomas and oncocytomas have to be considered for differential diagnosis based on their higher prevalence, while among malignant forms, carcinomas, represented by clear cell carcinomas in 70–80% of cases, papillary carcinomas in 14–17%, and chromophobe in 4–8% have to be considered [10].

In order to characterize small renal masses, many authors propose the imaging-guided biopsy, being able to predict the malignant nature of the lesion with a sensitivity ranging between 80% and 92% and a specificity ranging between 83% and 100% [11], with a low rate of serious complications (<1%) [12–15].

However, this method has some limitations because the sensitivity is reduced in lesions of <3 cm in diameter [11, 16], it does not provide information on any synchronous tumors, and it is not specific in the case of hybrid tumors and is contraindicated in patients with acquired polycystic disease [17].

With regard to the CT characterization, many interesting experiences have been reported in the literature, but the obtained results remain discordant by considering the different evaluation parameters which can be examined on unenhanced and enhanced scans [5–9].

The presence of intralesional calcifications represents a crucial diagnostic parameter being associated with carcinomas in most cases; in our experience, calcifications were found in 12.5% of cases, in agreement with data reported by Kim et al. [18]. On the contrary, in the study performed by Alshumrani et al. [19] calcifications were detected in 4 out of the 47 examined masses (8.5%), in particular in 3 carcinomas and in a case of inflammatory pseudotumor.

Regarding intralesional densitometry, negative HU values significant for the presence of adipose tissue were exclusively recognized in all the examined angiomyolipomas; therefore this finding, as reported by other authors [20], can be considered exhaustive for the characterization of the disease. However, it has to be considered that even in carcinomas the possibility of adipose tissue exists, but only in the case of bulky lesions [21, 22].

This fundamental diagnostic element could not be found in the lipid poor angiomyolipomas, which account for the 4-5% of cases; this kind of lesions could provide important problems for differential diagnosis with carcinomas or oncocytomas, but they did not occur in our series [18, 23].

With regard to the unenhanced intralesional densitometry, we found that clear cell carcinomas showed a mean higher density of 10 HU as compared to the contiguous renal parenchyma, chromophobe cell carcinomas of 4 HU, and papillary carcinomas 5 HU; on the contrary, oncocytomas had a mean slightly lower density of 4.5 HU. Despite our results, this element provides poor diagnostic information since it is reported in the literature that a hyperdense appearance can be recognized in such benign lesions as lipid poor angiomyolipomas, metanephric adenomas, leiomyomas, and oncocytomas and also in such malignant lesions as carcinomas and lymphomas [24].

On enhanced CT scans, no significant volumetric difference among oncocytomas, carcinomas, and angiomyolipomas was found in our series, in agreement with Millet et al. [15]; on the contrary, Frank et al. [25], in a review considering 2770 patients, reported that the smaller tumors were more frequently benign or otherwise in relation to lesions with low-grade malignancy.

With regard to the inhomogeneous morphology of the tumor, our results confirm those of Alshumrani et al. [19] who conclude their study arguing that the inhomogeneity of a mass cannot be considered a distinctive diagnostic element, as common to carcinomas and to oncocytomas, while they are in disagreement with those of Davidson et al. [26], who consider the presence of intralesional necrotic areas as highly suggestive of carcinomas.

In our experience, intralesional densitometry evaluated in the arterial, portal, and delayed phases has shown widely varying values in the same type of lesions and the possibility of overlapping values in different histological types occurred; therefore it has a poor diagnostic value in the characterization of the disease.

Only papillary carcinoma had a markedly lower enhancement after intravenous contrast medium injection as compared with all other lesions in our series, with mean values of 50.5 HU in the arterial phase, 60.1 HU in the portal phase, and 57.2 in the delayed phase; therefore, as confirmed by other authors [6, 27] papillary carcinoma represents the only subtype of renal lesion which can be characterized by considering this parameter.

With regard to the peak density, it was detected in the arterial phase (mean value, 183.5 HU) as compared to the portal phase (average value, 136 HU) in case of clear cell carcinomas; on the contrary, in case of chromophobe, papillary carcinomas, and oncocytomas, the peak was found in the portal phase as compared to the arterial one, with mean values of 210 HU versus 180 HU, 60 HU versus 50.3 HU, and 147.5 versus 114.5 HU, respectively.

These results, however, are not reflected in the literature, as in the study of Young et al. [14] clear cell carcinomas and oncocytomas showed a peak of enhancement in the arterial phase (resp., 125 HU and 106 HU) and in the study of Bird et al. [28] clear cell carcinomas showed no significant change between arterial and portal phases (110 HU and 108 HU, resp.), whereas oncocytomas showed a peak of enhancement in the arterial phase (171 HU). Finally Gakis et al. [29] found a peak of enhancement in the portal phase (resp., +67.5 and +48.5 HU as compared to the contiguous renal parenchyma) in case of carcinomas and oncocytomas.

Our results do not show significant evidence for differential diagnosis also with regard to the mean washout values, because of the possibility of substantially overlapping values in clear cell carcinomas, oncocytomas, and chromophobe carcinomas.

Only in case of papillary carcinomas and angiomyolipomas, significant different washout was found, with mean values of 2.7% and 50%, respectively.

These results are in disagreement with those reported by Bird et al. [28] who showed a washout of 83% (>50%) for the analyzed oncocytomas and therefore higher values as compared with carcinomas which showed a washout of less than 50%.

The “paradoxical effect” was described for the first time by Kim et al. [13] and it has been considered typical of oncocytoma (sensitivity of 80% and specificity of 99%); it was found in a single case of oncocytoma; therefore, in agreement with O'Malley et al. [17] such alteration is uncommon and in any case worthy of further investigation.

With regard to the diagnostic significance of the star-shaped scar, considered by Quinn et al. [27] as strongly suggestive of oncocytoma and recognized in 33% of cases, it did not occur in our series and therefore no assessment is allowed. However, it has been considered an uncommon finding in other studies and not significant for oncocytoma [8].

Finally, we evaluated the evidence of urinary tract infiltration and it was recognized only in 2/56 patients (3.6%). Although this is a significant element of malignant disease, as reported by other authors [30], it is rarely detected, because it is correlated to the tumor size and therefore unusual in renal carcinomas with a diameter of less than 4 cm.

Our study has important limitations mainly represented by the relatively small number of the enrolled patients, the consequent limited statistical evaluation, and the absence of a direct comparison between the different kinds of renal lesions.

## 5. Conclusions

CT has an excellent potential in recognizing small renal tumors, but its full accuracy in their characterization is still debated in the scientific literature.

Based on the reported experience, crucial elements in the unenhanced CT scans are represented by calcifications suggestive for carcinomas and adipose tissue for angiomyolipomas.

In enhanced CT scans, crucial elements are represented by the infiltration of the excretory tract indicative of malignant lesion and a modest increase in density associated with a reduced washout suggestive for papillary carcinomas.

The intralesional density values in the arterial, portal, and delayed phases, peak enhancement and washout values do not seem significant for differentiating clear cell, chromophobe carcinomas, angiomyolipomas, and oncocytomas. The diagnostic value of these elements requires further studies also considering the discrepancies reported in the literature.
